# A priori and a posteriori error analysis of the first order hyperbolic equation by using DG method

**DOI:** 10.1371/journal.pone.0277126

**Published:** 2023-03-30

**Authors:** Muhammad Shakhawat Hossain, Chunguang Xiong, Huafei Sun

**Affiliations:** Department of Mathematics, Beijing Institute of Technology, Beijing, China; Karlstad University: Karlstads Universitet, SWEDEN

## Abstract

In this research article, a discontinuous Galerkin method with a weighted parameter *θ* and a penalty parameter *γ* is proposed for solving the first order hyperbolic equation. The key aim of this method is to design an error estimation for both a priori and a posteriori error analysis on general finite element meshes. It is also exposed to the reliability and effectiveness of both parameters in the order of convergence of the solutions. For a posteriori error estimation, residual adaptive mesh- refining algorithm is employed. A series of numerical experiments are illustrated that demonstrate the efficiency of the method.

## 1 Introduction

The Discontinuous Galerkin method has been commonly used in industrial software packages for solving a large sort of computational fluid problem and numerical computation of partial differential equations. Discontinuous Galerkin (DG) method is a powerful tool for solving hyperbolic equations. The calculation of the problem is very ordinary, but it presents crucial numerical properties for solving more complicated problems. In 1973, Reed and Hill [1] introduced the Discontinuous Galerkin finite element method (DGFEM) by solving the neutron transport equation. Their results established better stability properties by comparing with the continuous Galerkin finite element method. The first appraisal of this method was showed in 1974 by Lesaint and Raviart [2]. Using more robust stability of DG scheme, this methods was later analyzed by Johnson, Navert, and J.Pitkäranta [3] and Johnson, J.Pitkäranta [4]. In 1988, Jonson [5] published an analysis of adaptive time-step error control of stiff ODEs for the DG method. Later on, in 1995, Estep [6] mingled this analysis with the general non-autonomous ODEs. Lastly, Böttcher and Rannacher [7] in 1996 used a new adaptive error control technique for solving ODEs by applying the DG method. By using an arbitrary shape of polygons meshes Mu and Ye [8] introduced a dissimilar scheme for solving first-order hyperbolic equations. Burman, Quarteroni and Stamm [9] developed an innovative format of interior penalty for both continuous and discontinuous finite element approximations of hyperbolic equations. [10, 11] proposed a technique for optimal order of convergence for some structured two-dimensional non-Cartesian grids and piecewise constant approximations. Then, Lin [12] reviewed this technique and showed a new error estimation of hyperbolic equations. Also, Xiong C, Luo F, Ma X, *et al*. in [13] derived an error analysis for DG finite element approximation governed by the first order linear hyperbolic equation. Finally, Burman and Stamm [14] proved a way of the optimal convergence grips for quadratic and higher polynomial degrees by penalizing the jump of the tangential part of the gradient. The least-squares finite element approximations for linear hyperbolic equations were introduced in [15–17]. [18–20] established a posteriori error estimator for the first-order linear hyperbolic equations. Moreover, Houston, Schwab and Süli [21] stabilized the finite element approximation of the hp-version of both streamline-diffusion(SD) and DG methods, while, Xiong and Li [22] improved a posteriori error analysis for the optimal control problems of the first-order linear hyperbolic equation. Brezzi, Marini and Süli [23] extended the upwind method by changing the standard upwind flux with a reliability term and a jump stabilization term. Based on numerical fluxes, the discontinuous Galerkin method for a hyperbolic equation had significant progress in [24]. An extensive literature was made to develop the DG methods for hyperbolic problems in [25–32]. The objective of this article is to explore an error estimation for the first order hyperbolic equation by using Discontinuous Galerkin (DG) finite element method. The key aim of this article is to design an appropriate error estimates by using spacial stability parameters such as a weighted parameter *θ* and a penalty parameter *γ* that presents a guider on the order of convergence of the solutions. It is considered the penalty function *τ* > 0 as $\tau =\frac{\theta |\beta \cdot n|}{h^{\gamma }}.$ For a specific value of these two parameters it can be recovered from the classical discontinuous Galerkin method. More explicitly, the method is recovered from [23] by choosing *γ* = 1 and from the classical upwind scheme with *θ* = 1. It is also Privileged to point out that, it is a different and simpler approach to seek an error analysis from all the finite element schemes for linear hyperbolic equations including the methods cited here. The rest of the paper is structured as follows. A brief review of the problem governed by first order hyperbolic equations, construction of the DG finite element method and discussion of the stability analysis of the method are presented in section 2. Section 3 and Section 4, derive the a priori and a posteriori error estimates of the problems and show the convergence analysis of DGFEM methods. A number of numerical examples are offered in section 5 to demonstrate the theoretical results.

## 2 Model problem and discretization

The objective of this section is to state the problem in details, introduce the finite element meshes and space, and also putting on some others preliminary results. The objective of this section are to state the problem in details, introduce the finite element meshes and space and establish some other preliminary results.

### 2.1 Formulation of the problem

A first order hyperbolic equation is defined in a polyhedral bounded domain Ω in $R^{d}\left(d=2,3\right)$ and Γ be the union of it’s (*d* − 1) -dimensional open faces. To seek an unknown function *u* = *u*(*x*) consider the following hyperbolic boundary value problem:

$$\left\{ \begin{array}{c} \beta \cdot \nabla u+bu=f\;in\Omega ,\\ {u|}_{\Gamma_{-}}=g\;on\;\partial \Omega . \end{array}\right.$$ (1)

Where, *β* = (*β*_1_, *β*_2_, ……, *β*_*d*_) is a *d*− component vector function defined on Ω with $\beta_{i}\in C^{1}\left(\bar{\Omega }\right),i=1,2,....,d$ and $b\in C(\bar{\Omega })$. Again, *f* ∈ *L*^2^(Ω) and *g* ∈ *L*^2^(Γ_−_) are assumed to be sufficiently smooth real-valued functions, which could be different in different problems.

Define the following subsets of Γ = *∂*Ω:

$$\Gamma_{-}=\left\{x\in \Gamma :\beta \left(x\right)\cdot n\left(x\right)<0\right\},$$

$$\Gamma_{+}=\left\{x\in \Gamma :\beta \left(x\right)\cdot n\left(x\right)\geq 0\right\}.$$

They are known as inflow boundary and outflow boundary, respectively and **n**(*x*) is the unit outward normal vector to Γ at *x* ∈ *∂*Ω. Consequently, Γ_−_ and Γ_+_ are not necessarily connected subset of Γ as well as Γ is non-characteristic in the sense that Γ_−_ ⋃ Γ_+_ = Γ. The control space *Y* ∈ *L*^2^(Ω), and *k* is a closed convex subset of *Y*. In addition, there exists a vector *ξ*_1_ ∈ *R*^*d*^ such that

$$\begin{array}{c} b\left(x\right)+\frac{1}{2}\nabla \cdot \beta \left(x\right)+\beta \left(x\right)\cdot \xi_{1}\geq 0\;a.e.\;x\in \Omega . \end{array}$$ (2)

For simplicity of (2), assume *ξ*_1_ = 0 in the above hypothesis, then define the positive constant *c*_0_ by,

$$\begin{array}{c} b\left(x\right)+\frac{1}{2}\nabla \cdot \beta \left(x\right)\geq c_{0}>0\;a.e.\;x\in \Omega . \end{array}$$ (3)

The operator *b* is linear continuous operator and b* is the adjoin of *b* then, assume that the condition holds for every constant *c* such that,

$$\begin{array}{c} \left|\left(bu,v\right)|=\right|\left(u,b^{*}v\right)|\leq c\|u\|\|v\|\;u,v\in Y. \end{array}$$

### 2.2 Finite element mesh and space

The approximation of (1) will be done by using discontinuous finite elements to construct finite element meshes over a convex polygons Ω. Consider a family of partition $T_{h}$ into disjoin open regular of the domain Ω and denote by ${\left(T_{h}\right)}_{h>0}$ is an affine shape-regular or 1-irregular mesh sequence. The size and shape of an element *K* of $T_{h}$ are measured in terms of two quantities, *h*_*K*_ and *ρ*_*K*_, defined as:

$$h_{K}=diam\left(K\right),$$

$$\rho_{K}=\text{sup}\left\{diam\left(B\right),B\;is\;a\;ball\;contained\;in\;the\;element\;K\right\},$$

i.e., the mesh parameter *h*_*K*_ is defined as a cell wise constant function by setting *h*|_*K*_ = *h*_*K*_, and *h*_*K*_ is the diameter of *K*. There exists two constants *C*_1_ and *C*_2_ such that the quantities should satisfy

$$C_{1}\leq \frac{h_{K}}{\rho_{K}}\leq C_{2}.$$

Also, the mesh $T_{h}$ are combination of triangles *K* with the diameter *h*_*K*_ and the mesh size of $h=max_{K\in T_{h}}h_{K}$. For each triangulation ${\left(T_{h}\right)}_{h>0}$, introduce *V*_*h*_ to denote the corresponding discontinuous finite element space of the piecewise polynomial $P_{k}\left(K\right)$ with the degree *k* ≥ 1 as follows:

$$V_{h}=\left\{v\left(t\right)\in L^{2}\left(\Omega \right):v{|}_{K}\in P_{k}\left(K\right),\forall K\in T_{h}\right\}.$$

Again, for a given $K\in T_{h}$, denote the non-standard notation by *∂* *K*, the union of (*d* − 1)-dimensional open faces of *K* as well as the subset of the boundary of *K*. Let *x* ∈ *∂* *K* and *n*(*x*) denotes the unit outward normal vector to *∂* *K* at *x*. With these assumptions, define the inflow and outflow parts of *∂* *K*, respectively, by

$$\begin{array}{c} \partial K_{-}=\left\{x\in \partial K:\beta \left(x\right)n\left(x\right)<0\right\},\\ \partial K_{+}=\left\{x\in \partial K:\beta \left(x\right)n\left(x\right)\geq 0\right\}. \end{array}$$

Here, *v*^+^ denotes the interior trace on *∂* *K* (the trace taken from within *K*). If *∂* *K*_−_∖Γ_−_ is nonempty for some *K* in $T_{h}$, then define the outer trace *v*^−^ of *v* on *∂* *k*_−_∖Γ_−_. When *x* ∈ Γ, define the inner trace *v*^+^ = *v*(*x*), and outer trace *v*^−^ = 0. So, it may defines the jump and average of *v* across the edge of the element *K*:

$${\left[v\right]|}_{\partial K}=v^{+}-v^{-},$$

$${\left\{v\right\}|}_{\partial K}=\frac{1}{2}\left(v^{+}+v^{-}\right).$$

Now decomposing *∂* *K* into union of four disjoint parts

$$\partial K=\left(\partial K_{-}\cap \Gamma_{-}\right)\cup \left(\partial K_{-}\backslash \Gamma_{-}\right)\cup \left(\partial K_{+}\cap \Gamma_{+}\right)\cup \left(\partial K_{+}\backslash \Gamma_{+}\right).$$

Then, in order to construct a weak formulation for the problem (1), introduce a bilinear form *a*(*u*_*h*_, *v*) for $a\left(u_{h},v\right):\left(V_{h}\times V_{h}\right)\to R$ as

$$\begin{array}{c} \begin{array}{cc} a(u_{h},v)= & -\sum_{K\in T_{h}}{\left(u_{h},\beta \nabla v\right)}_{K}+\sum_{K\in T_{h}}{\left(bu_{h},v\right)}_{K}+\sum_{K\in T_{h}}\int_{\partial K_{-}\backslash \Gamma_{-}}\left(\left(\beta \cdot n\right)\left\{u_{h}\right\}+\tau \left[u_{h}\right]\right)\left[v\right]ds\\ + & \sum_{K\in T_{h}}\int_{\partial K_{-}\cap \Gamma_{-}}\left(\beta \cdot n\right)u_{h}^{+}v^{+}ds. \end{array} \end{array}$$ (4)

Therefore, the finite element method is to find *u*_*h*_ ∈ *V*_*h*_ satisfying

$$\begin{array}{c} a\left(u_{h},v\right)={\left(f,v\right)}_{K}-\sum_{K\in T_{h}}\int_{\partial K_{-}\cap \Gamma_{-}}\left(\beta \cdot n\right)gv^{+}ds,\;\;\forall v\in V_{h}. \end{array}$$ (5)

The above scheme yield system of differential equation. Since the Stiffness matrix associated with (*u*_*h*_, *v*) is positive definite. Therefore, the system is uniquely solvable for a consistent initial condition. Now, the boundary inner product and the norm associated with the inner product are defined as.

$${\left(u,v\right)}_{\partial K}=\int_{\partial K}\left(\beta \cdot n\right)uvds,$$

$${\left\|u\right\|}_{\partial K}^{2}=\int_{\partial K}\left|\left(\beta \cdot n\right)\right|u^{2}ds.$$

As well as the following DG-norm is defined by

$$\begin{array}{ccc} \left\|\right|u_{h}{\left|\right\|}_{DG}^{2} & = & c_{0}\sum_{K\in T_{h}}\|u_{h}{\|}_{K}^{2}+\sum_{K\in T_{h}}\tau \|u_{h}^{+}-u_{h}^{-}{\|}_{\partial K_{-}\backslash \Gamma_{-}}^{2} \end{array}$$

$$\begin{array}{ccc} & & +\frac{1}{2}\sum_{K\in T_{h}}\|u_{h}^{+}{\|}_{\partial K_{+}\cap \Gamma_{+}}^{2}+\frac{1}{2}\sum_{K\in T_{h}}\|u_{h}^{+}{\|}_{\partial K_{-}\cap \Gamma_{-}}^{2}. \end{array}$$

Thus, the following lemma is introduced for the stability analysis of this method.

**Lemma 2.1** *Assume that there exists a positive constant* *c*_0_ *such that* (3) *holds. Then the solution* *u*_*h*_ *satisfies the following bound*

$$\begin{array}{ccc} & & c_{0}\sum_{K\in T_{h}}\|u_{h}{\|}_{K}^{2}+\sum_{K\in T_{h}}\tau \|u_{h}^{+}-u_{h}^{-}{\|}_{\partial K_{-}\backslash \Gamma_{-}}^{2}+\sum_{K\in T_{h}}\|u_{h}^{+}{\|}_{\partial K_{+}\cap \Gamma_{+}}^{2}\\ & & +\sum_{K\in T_{h}}\|u_{h}^{+}{\|}_{\partial K_{-}\cap \Gamma_{-}}^{2}\leq \frac{1}{c_{0}}\sum_{K\in T_{h}}\|f{\|}_{K}^{2}+2\sum_{K\in T_{h}}\|g{\|}_{\partial K_{-}\backslash \Gamma_{-}}^{2}. \end{array}$$

*Proof*: The Eq (5) can be rewritten by considering *v* = *u*_*h*_.

$$\begin{array}{c} a\left(u_{h},u_{h}\right)=\left(f,u_{h}\right)-\sum_{e\in \Gamma_{-}}\int_{e}\left(\beta \cdot n\right)gu_{h}^{+}ds,\;\;\forall u_{h}\in V_{h}. \end{array}$$ (6)

Therefore, the left hand side of Eq (6) can be bounded by,

$$\begin{array}{c} \begin{array}{cc} & a(u_{h},u_{h})\\ = & -\sum_{K\in T_{h}}{\left(u_{h},\beta \nabla u_{h}\right)}_{K}+\sum_{K\in T_{h}}{\left(bu_{h},u_{h}\right)}_{K}+\sum_{K\in T_{h}}\int_{\partial K_{-}\backslash \Gamma_{-}}\left(\left(\beta \cdot n\right)\left\{u_{h}\right\}+\tau \left[u_{h}\right]\right)\left[u_{h}\right]ds\\ & +\sum_{K\in T_{h}}\int_{\partial K_{-}\cap \Gamma_{-}}\left(\beta \cdot n\right)u_{h}u_{h}ds\\ = & \sum_{K\in T_{h}}\int_{K}\left(b+\frac{\nabla \cdot \beta }{2}\right)|u_{h}{|}^{2}dx-\frac{1}{2}\sum_{K\in T_{h}}\int_{\partial K}|\left(\beta \cdot n\right)||u_{h}^{+}{|}^{2}ds\\ & +\sum_{K\in T_{h}}\int_{\partial K_{-}\backslash \Gamma_{-}}\left(\left(\beta \cdot n\right)\left\{u_{h}\right\}+\tau \left[u_{h}\right]\right)\left[u_{h}\right]ds+\sum_{K\in T_{h}}\int_{\partial K_{-}\cap \Gamma_{-}}\left(\beta \cdot n\right)u_{h}u_{h}ds. \end{array} \end{array}$$ (7)

Now,

$$\begin{array}{c} \begin{array}{cc} & \frac{1}{2}\sum_{K\in T_{h}}\int_{\partial K}\left|\left(\beta \cdot n\right)\right||u_{h}^{+}{|}^{2}ds\\ = & \frac{1}{2}\sum_{K\in T_{h}}\int_{\partial K_{-}\backslash \Gamma_{-}}\left|\left(\beta \cdot n\right)\right||u_{h}^{+}{|}^{2}ds+\frac{1}{2}\sum_{K\in T_{h}}\int_{\partial K_{-}\cap \Gamma_{-}}\left|\left(\beta \cdot n\right)\right||u_{h}^{+}{|}^{2}ds\\ & +\frac{1}{2}\sum_{K\in T_{h}}\int_{\partial K_{+}\cap \Gamma_{+}}\left|\left(\beta \cdot n\right)\right||u_{h}^{+}{|}^{2}ds+\frac{1}{2}\sum_{K\in T_{h}}\int_{\partial K_{+}\backslash \Gamma_{+}}\left|\left(\beta \cdot n\right)\right||u_{h}^{+}{|}^{2}ds. \end{array} \end{array}$$ (8)

Again,

$$\begin{array}{c} \begin{array}{cc} & \sum_{K\in T_{h}}\int_{\partial K_{-}\backslash \Gamma_{-}}\left(\beta \cdot n\right)\left\{u_{h}\right\}\left[u_{h}\right]ds+\sum_{K\in T_{h}}\int_{\partial K_{-}\backslash \Gamma_{-}}\tau \left[u_{h}\right]\left[u_{h}\right]ds\\ = & \sum_{K\in T_{h}}\int_{\partial K_{-}\backslash \Gamma_{-}}\left(\beta \cdot n\right)\frac{1}{2}\left(u_{h}^{+}+u_{h}^{-}\right)\left(u_{h}^{+}-u_{h}^{-}\right)ds+\sum_{K\in T_{h}}\int_{\partial K_{-}\backslash \Gamma_{-}}\tau {\left(u_{h}^{+}-u_{h}^{-}\right)}^{2}ds\\ = & \frac{1}{2}\sum_{K\in T_{h}}\int_{\partial K_{-}\backslash \Gamma_{-}}\left(\beta \cdot n\right)|u_{h}^{+}{|}^{2}ds-\frac{1}{2}\sum_{K\in T_{h}}\int_{\partial K_{-}\backslash \Gamma_{-}}\left(\beta \cdot n\right){\left|u_{h}^{-}\right|}^{2}ds\\ & +\sum_{K\in T_{h}}\int_{\partial K_{-}\backslash \Gamma_{-}}\tau {\left(u_{h}^{+}-u_{h}^{-}\right)}^{2}ds. \end{array} \end{array}$$ (9)

Also, according to the hypothesis used in [21],

$$\begin{array}{c} \begin{array}{c} \frac{1}{2}\sum_{K\in T_{h}}\int_{\partial K_{+}\backslash \Gamma_{+}}\left(\beta \cdot n\right)|u_{h}^{+}{|}^{2}ds+\frac{1}{2}\sum_{K\in T_{h}}\int_{\partial K_{-}\backslash \Gamma_{-}}\left(\beta \cdot n\right){\left|u_{h}^{-}\right|}^{2}ds=0. \end{array} \end{array}$$ (10)

Inserting Eqs (8), (9) and (10) into Eq (7) and using Eq (3), it is found that

$$\begin{array}{c} \begin{array}{cc} a(u_{h},u_{h})\geq & c_{0}\sum_{K\in T_{h}}\|u_{h}{\|}_{K}^{2}+\frac{1}{2}\sum_{K\in T_{h}}{\left\|u_{h}^{+}\right\|}_{\partial K_{+}\cap \Gamma_{+}}^{2}\\ + & \frac{1}{2}\sum_{K\in T_{h}}\|u_{h}^{+}{\|}_{\partial K_{-}\cap \Gamma_{-}}^{2}+\sum_{K\in T_{h}}\tau {\left\|u_{h}^{+}-u_{h}^{-}\right\|}_{\partial K_{-}\backslash \Gamma_{-}}^{2}. \end{array} \end{array}$$ (11)

The right hand side of (6) can be bounded by using Cauchy-Schwarz and Young inequalities

$$\begin{array}{c} \begin{array}{c} |\left(f,u_{h}\right)-\sum_{e\in \Gamma_{-}}\int_{e}\left(\beta \cdot n\right)gu_{h}^{+}ds|\leq \sum_{K\in T_{h}}{\left\|f\right\|}_{k}\|u_{h}{\|}_{k}+\sum_{K\in T_{h}}{\left\|g\right\|}_{\partial K_{-}\backslash \Gamma_{-}}{\left\|u_{h}^{+}\right\|}_{\partial K_{-}\backslash \Gamma_{-}}\\ \leq \frac{c_{0}}{2}\sum_{K\in T_{h}}\|u_{h}{\|}_{k}^{2}+\frac{1}{2c_{0}}\sum_{K\in T_{h}}{\left\|f\right\|}_{k}^{2}+\frac{1}{4}\sum_{K\in T_{h}}\|u_{h}^{+}{\|}_{\partial K_{-}\backslash \Gamma_{-}}^{2}+\sum_{K\in T_{h}}{\left\|g\right\|}_{\partial K_{-}\backslash \Gamma_{-}}^{2}. \end{array} \end{array}$$ (12)

The proof of this lemma can be completed by using Eqs (11) and (12).

## 3 A priori error estimate

In this section, the discussion of the a prior error analysis of the problem governed by the first order hyperbolic Eq (1) is given. For the analysis, assume the problem (1) satisfy the following assumptions:

$$\begin{array}{c} \beta \nabla_{T_{h}}v_{h}\in V_{h}\left(\Omega ,T_{h}\right)\;\forall v_{h}\in V_{h}\left(\Omega ,T_{h}\right),\\ b\in V_{h}\left(\Omega ,T_{h}\right),\;f\in V_{h}\left(\Omega ,T_{h}\right). \end{array}$$

Here, $\nabla_{T_{h}}v,v\in H^{1}\left(\Omega ,T_{h}\right)$ denotes the broken gradient of *v* defined by $\left(\nabla_{T_{h}}v\right){|}_{k}=\nabla \left(v{|}_{k}\right),k\in T_{h}$. For the significant of (1), we also assume that $\beta \in {\left[W_{\infty }^{1}\left(\Omega \right)\right]}^{d}.$

For the purpose of a priori error estimate, the following lemmas in [21, 23] are introduced for the finite element space *V*_*h*_.

**Lemma 3.1** *Given v* ∈ *V*_*h*_ *there exists a positive constant C, dependent only on d and the shape-regularity of* $T_{h}$, *such that*

$${\left|v\right|}_{H^{1}\left(K\right)}\leq \frac{C}{h_{K}}\|v{\|}_{L_{2}\left(K\right)}\;\forall K\;in\;T_{h}.$$

On the other hand, the choice of projection operator is essential in the a priori error estimate. Thus, the following lemma is crucial for this analysis.

**Lemma 3.2** *Let us denote by* Π *be the L*^2^-*projector onto the finite element space V*_*h*_, *i.e., given that u* ∈ *L*^2^(Ω), *define* Π, *for which the following standard estimate holds*

$$\begin{array}{c} \|u-{\Pi u\|}_{r,T}\leq Ch^{k+1-r}{\left\|u\right\|}_{k+1,T},r=0,1,T\in T_{h}. \end{array}$$

*Again, If e is common edge of two triangles then recall the following trace inequality*

$$\begin{array}{c} \|u-{\Pi u\|}_{0,e}^{2}\leq {C(|e|}^{-1}\|u-{\Pi u\|}_{0,T}+|e\||u-{\Pi u\|}_{1,T}), \end{array}$$

*with C a positive constant depending only on the minimum angle of T. Thus, from above two equations, it can deduced that*

$$\begin{array}{c} \|u-{\Pi u\|}_{L^{2}\left(\Omega \right)}\leq Ch^{r+1}{\left\|u\right\|}_{H^{r}\left(\Omega \right)}. \end{array}$$ (13)

Now, the following theorem is proposed for the analysis of the a priori error estimate of the problem governed by the Eq (1).

**Theorem 3.1** *Let u*_*h*_ ∈ *V*_*h*_ *be the finite element solution of* (1) *arising from* (5). *Then there exists a constant C such that*:

$$\begin{array}{cc} \|u-u_{h}{\|}_{DG}^{2}\leq & C\sum_{K}h_{K}^{2r+1}{\left\|u\right\|}_{H^{r}\left(K\right)}^{2}. \end{array}$$

*Proof*: The error *u* − *u*_*h*_ satisfies the following equation

$$\begin{array}{c} a\left(u-u_{h},v_{h}\right)=0,\forall v_{h}\in V_{h}.\\ a(u-\Pi u+\Pi u-u_{h},v_{h})=0, \end{array}$$ (14)

Take Π*u* − *u*_*h*_ = *ξ* and *u* − Π*u* = *ζ* where Π is a suitable projection. Now, put *v*_*h*_ = *ξ* and use Cauchy-Schwarze inequality in (14), to get

$$\begin{array}{ccc} {\left\|\xi \right\|}_{DG}^{2} & \leq & a(\xi ,\xi )=|a(\zeta ,\xi )|. \end{array}$$

$$\begin{array}{ccc} a(\zeta ,\xi ) & = & -\sum_{K}{\left(\zeta ,\beta \nabla \xi \right)}_{K}+\sum_{K}{\left(b\zeta ,\xi \right)}_{K}+\sum_{K}\int_{\partial K_{-}\backslash \Gamma_{-}}\left(\left(\beta \cdot n\right)\left\{\xi \right\}+\tau \left[\xi \right]\right)\left[\zeta \right]ds\\ & & +\sum_{K}\int_{\partial K_{-}\cap \Gamma_{-}}\left(\beta \cdot n\right)\zeta^{+}\xi^{+}ds\\ & = & \sum_{K}{\left(\left(b+\nabla \cdot \beta \right)\zeta ,\xi \right)}_{K}-\sum_{K}\int_{\partial K}\left(\beta \cdot n\right)\zeta^{+}\xi^{+}ds+\sum_{K}\int_{\partial K_{-}\backslash \Gamma_{-}}\left(\beta \cdot n\right)\left\{\xi \right\}\left[\zeta \right]ds\\ & & +\sum_{K}\int_{\partial K_{-}\backslash \Gamma_{-}}\tau \left[\xi \right]\left[\zeta \right]ds+\sum_{K}\int_{\partial K_{-}\cap \Gamma_{-}}\left(\beta \cdot n\right)\zeta^{+}\xi^{+}ds\\ & = & \sum_{K}{\left(\left(b+\nabla \cdot \beta \right)\zeta ,\xi \right)}_{K}-\sum_{K}\int_{\partial K_{-}\backslash \Gamma_{-}}\left(\beta \cdot n\right)\zeta^{+}\xi^{+}ds\\ & & -\sum_{K}\int_{\partial K_{+}\backslash \Gamma_{+}}\left(\beta \cdot n\right)\zeta^{+}\xi^{+}ds-\sum_{K}\int_{\partial K_{-}\cap \Gamma_{-}}\left(\beta \cdot n\right)\zeta^{+}\xi^{+}ds\\ & & -\sum_{K}\int_{\partial K_{+}\cap \Gamma_{+}}\left(\beta \cdot n\right)\zeta^{+}\xi^{+}ds+\sum_{K}\int_{\partial K_{-}\backslash \Gamma_{-}}\left(\beta \cdot n\right)\left\{\xi \right\}\left[\zeta \right]ds\\ & & +\sum_{K}\int_{\partial K_{-}\backslash \Gamma_{-}}\tau \left[\xi \right]\left[\zeta \right]ds+\sum_{K}\int_{\partial K_{-}\cap \Gamma_{-}}\left(\beta \cdot n\right)\zeta^{+}\xi^{+}ds. \end{array}$$

Then, $\sum_{K}\int_{\partial K_{+}\backslash \Gamma_{+}}\left(\beta \cdot n\right)\zeta^{+}\xi^{+}ds=-\sum_{K}\int_{\partial K_{-}\backslash \Gamma_{-}}\left(\beta \cdot n\right)\zeta^{-}\xi^{-}ds,$ and use the identity *a*^+^*b*^+^ − *a*^−^*b*^−^ − {*a*}[*b*] = [*a*]{*b*}, It can be calculated that,

$$\begin{array}{c} \begin{array}{cc} a(\zeta ,\xi )= & \sum_{K}{\left(\left(b+\nabla \cdot \beta \right)\zeta ,\xi \right)}_{K}+\sum_{K}\int_{\partial K_{-}\backslash \Gamma_{-}}\tau \left[\xi \right]\left[\zeta \right]ds-\sum_{K}\int_{\partial K_{+}\cap \Gamma_{+}}\left(\beta \cdot n\right)\zeta^{+}\xi^{+}ds\\ & -\sum_{K}\int_{\partial K_{-}\backslash \Gamma_{-}}\left(\beta \cdot n\right)\left[\xi \right]\left\{\zeta \right\}ds. \end{array} \end{array}$$

Applying Eq (3)

$$\begin{array}{c} \begin{array}{cc} a(\zeta ,\xi )\leq & \sum_{K}{\left(c_{0}\zeta ,\xi \right)}_{K}+\sum_{K}\int_{\partial K_{-}\backslash \Gamma_{-}}\tau \left[\xi \right]\left[\zeta \right]ds-\sum_{K}\int_{\partial K_{+}\cap \Gamma_{+}}\left(\beta \cdot n\right)\zeta^{+}\xi^{+}ds\\ & -\sum_{K}\int_{\partial K_{-}\backslash \Gamma_{-}}\left(\beta \cdot n\right)\left[\xi \right]\left\{\zeta \right\}ds. \end{array} \end{array}$$ (15)

By using Young inequality in (15), it can be found that

$$\begin{array}{c} \begin{array}{cc} a(\zeta ,\xi )\leq & \frac{1}{2}\sum_{K}\|c_{0}{\xi \|}_{K}^{2}+2\sum_{K}\|c_{0}{\zeta \|}_{K}^{2}+\frac{1}{2}\sum_{K}{\left\|\left[\xi \right]\right\|}_{\partial K_{-}\backslash \Gamma_{-}}^{2}+2\sum_{K}{\left\|\left\{\zeta \right\}\right\|}_{\partial K_{-}\backslash \Gamma_{-}}^{2}\\ & +\frac{1}{4}\sum_{K}\|\xi^{+}{\|}_{\partial K_{+}\cap \Gamma_{+}}^{2}+\sum_{K}\|\zeta^{+}{\|}_{\partial K_{+}\cap \Gamma_{+}}^{2}+\frac{1}{4}\sum_{K}{\tau \|\left[\xi \right]\|}_{\partial K_{-}\backslash \Gamma_{-}}^{2}+\sum_{K}\tau {\left\|\left[\zeta \right]\right\|}_{\partial K_{-}\backslash \Gamma_{-}}^{2}. \end{array} \end{array}$$

Therefore,

$$\begin{array}{c} \begin{array}{cc} {\left\|\xi \right\|}_{DG}^{2}\leq & \sum_{K}\|c_{0}{\zeta \|}_{K}^{2}+\sum_{K}{\left\|\left\{\zeta \right\}\right\|}_{\partial K_{-}\backslash \Gamma_{-}}^{2}+\sum_{K}\|\zeta^{+}{\|}_{\partial K_{+}\cap \Gamma_{+}}^{2}+\sum_{K}\tau {\left\|\left[\zeta \right]\right\|}_{\partial K_{-}\backslash \Gamma_{-}}^{2}. \end{array} \end{array}$$

Again,

$$\begin{array}{c} \begin{array}{cc} \|u-u_{h}{\|}_{DG}^{2}\leq & {\left\|\xi \right\|}_{DG}^{2}+{\left\|\zeta \right\|}_{DG}^{2}\\ \leq & {\left\|\zeta \right\|}_{DG}^{2}+\sum_{K}\|c_{0}{\zeta \|}_{K}^{2}+\sum_{K}{\left\|\left\{\zeta \right\}\right\|}_{\partial K_{-}\backslash \Gamma_{-}}^{2}+\sum_{K}\|\zeta^{+}{\|}_{\partial K_{+}\cap \Gamma_{+}}^{2}+\sum_{K}\tau {\left\|\left[\zeta \right]\right\|}_{\partial K_{-}\backslash \Gamma_{-}}^{2}\\ \leq & 2c_{0}\sum_{K}{\left\|\zeta \right\|}_{K}^{2}+\frac{1}{2}\sum_{K}{\left\|\left[\zeta \right]\right\|}_{\partial K_{-}\backslash \Gamma_{-}}^{2}+\sum_{K}{\left\|\left\{\zeta \right\}\right\|}_{\partial K_{-}\backslash \Gamma_{-}}^{2}+\frac{3}{2}\sum_{K}{\left\|\zeta^{+}\right\|}_{\partial K_{+}\cap \Gamma_{+}}^{2}\\ & +2\sum_{K}{\tau \|\left[\zeta \right]\|}_{\partial K_{-}\backslash \Gamma_{-}}^{2}+\frac{1}{2}\sum_{K}{\left\|\zeta^{+}\right\|}_{\partial K_{-}\cap \Gamma_{-}}^{2}. \end{array} \end{array}$$

Select *ζ* = *u* − Π*u* and by applying lemma 3.1 and lemma 3.2, it is calculated that

$$\begin{array}{c} \|u-u_{h}{\|}_{DG}^{2}\leq C\sum_{K}h_{K}^{2r+1}{\left\|u\right\|}_{H^{r}\left(K\right)}^{2}. \end{array}$$

Finally, note that, the constant extensively depends upon stability function *τ* and the results almost similar to the result of [23]. Therefore, this theorem shows the efficiency of parameters *θ* and *γ* upon the order of convergence of the error.

## 4 A posteriori error estimate

It frequently happens in practical problems that, due to the character of the data in specific sub-domains, a solution of a boundary value problem is less regular. In this case, without using too many additional degrees of freedom, it would be better to increase the accuracy of the Finite element approximation. Adaptive techniques based on a posteriori error estimators have become a crucial tool and are well recognized for such methods. For the a posteriori error estimate, a residual-based error estimate method is employed. For the mesh adaption and error estimates, consider the classical residual error indicator *η*_*K*_ on the element *K* for the problem. The local error indicator *η*_*K*_ is define as follows:

$$\begin{array}{c} \begin{array}{cc} \eta_{K}^{2}= & \sum_{K\in T_{h}}h_{K}^{2}\|f-div\left(\beta u_{h}\right)-bu_{h}{\|}_{L^{2}\left(K\right)}^{2}+\sum_{e\in \partial K_{-}\backslash \Gamma_{-}}h_{e}\tau \|\left[u_{h}\right]{\|}_{L^{2}\left(e\right)}^{2}\\ + & \sum_{e\in \partial K_{+}\cap \Gamma_{+}}h_{e}\|u_{h}{\|}_{L^{2}\left(e\right)}^{2}+\sum_{e\in \partial K_{-}\cap \Gamma_{-}}h_{e}\|g-u_{h}{\|}_{L^{2}\left(e\right)}^{2}. \end{array} \end{array}$$

Where, *h*_*K*_ is the longest’s edge of *K* and *h*_*e*_ is the length of edge *e*. Summing up the squares over all triangles to get a global quantity of the residual error indicator *η*.

$$\eta =\sum_{K\in T_{h}}{\left(\eta_{K}\right)}^{\frac{1}{2}}.$$

Now, for adapting the mesh, the new mesh size is given by the following formulae:

$$h_{n+1}\left(x\right)=\frac{h_{n}\left(x\right)}{f_{n}\left(\eta_{K}\left(x\right)\right)},$$

where, *η*_*K*_(*x*) is the local error indicator at point *x*, *h*_*n*_(*x*) is the previous mesh size and *f*_*n*_ is a user function defined by *f*_*n*_ = *min*(3, *max*(1/3, *η*_*K*_/η*)). The η* is defined by η* = *mean*(*η*_*K*_)*c*, where, *c* is an user coefficient generally closed to one at a certain partition.

Now, the following two theorems are proposed for the discussion about the a posteriori error estimates of the problem governed by the Eq (1).

**Theorem 4.1** *Let u and u*_*h*_ ∈ *V*_*h*_ *be the finite element solutions of* (1) *and* (5), *respectively. For any* 0 < *e* ≤ *K* *and h* > 0, *there is a constant C which is dependent on* $T_{h}$ *but independent of u*, *e and h*. *Then for the upper bound the following error representation formula holds*:

$$\begin{array}{c} \|u-u_{h}{\|}_{L^{2}\left(\Omega \right)}\leq C\eta . \end{array}$$

*Proof*: Let, *β*∇*v* ∈ *L*^2^(Ω), then, by using integration by part,

$$\begin{array}{ccc} \left(u-u_{h},\beta \nabla v\right) & = & \sum_{K}\int_{\partial K}\left(\beta \cdot n\right)\left(u-u_{h}\right)vds+\sum_{K}{\left(div\left(\beta \left(u-u_{h}\right)\right),v\right)}_{K}\\ & = & \sum_{K}\int_{\partial K}\left(\beta \cdot n\right)\left(u-u_{h}\right)vds+\sum_{K}(div{\left(\beta \left(u-u_{h}\right),v-v_{h}\right)}_{K}\\ & & +\sum_{K}{\left(div\left(\beta u_{h}\right),v_{h}\right)}_{K}\\ & = & \sum_{K}(div{\left(\beta \left(u-u_{h}\right),v-v_{h}\right)}_{K}+\sum_{K}\int_{\partial K}\left(\beta \cdot n\right)\left(u-u_{h}\right)vds\\ & & +\sum_{K}\int_{\partial K}\left(\beta \cdot n\right)u_{h}v_{h}ds-\sum_{K}{\left(\beta u_{h},\nabla v_{h}\right)}_{K}\\ & = & \sum_{K}(div{\left(\beta \left(u-u_{h}\right),v-v_{h}\right)}_{K}+\sum_{K}\int_{\partial K}\left(\beta \cdot n\right)\left(u-u_{h}\right)vds\\ & & +\sum_{K}\int_{\partial K}\left(\beta \cdot n\right)u_{h}v_{h}ds-\sum_{K}\int_{\partial K_{-}\backslash \Gamma_{-}}\left(\beta \cdot n\right)\left\{u_{h}\right\}+\tau \left[u_{h}\right])\left[v_{h}\right]ds\\ & & -\sum_{K}{\left(bu_{h},v_{h}\right)}_{K}-\int_{\partial K_{-}\cap \Gamma_{-}}\left(\beta \cdot n\right)u_{h}^{+}v_{h}^{+}ds+\sum_{K}{\left(f,v_{h}\right)}_{K}\\ & & -\int_{\partial K_{-}\cap \Gamma_{-}}\left(\beta \cdot n\right)gv_{h}^{+}ds\\ & = & \sum_{K}{\left(f-div\left(\beta u_{h}\right)-bu_{h},v-v_{h}\right)}_{K}+\int_{\partial K_{+}\cap \Gamma_{+}}\left(\beta \cdot n\right)u_{h}\left(v_{h}-v\right)ds\\ & & +\int_{\partial K_{-}\backslash \Gamma_{-}}\left(\tau \left[u_{h}\right]\left[v_{h}-v\right]+\left(\beta \cdot n\right)\left[u_{h}\right]\left\{v-v_{h}\right\}\right)ds\\ & & +\int_{\partial K_{-}\cap \Gamma_{-}}\left(\beta \cdot n\right)\left(g-u_{h}\right)\left(v_{h}-v\right)ds. \end{array}$$

Choosing *v*_*h*_ = Π_*h*_*u*, where, the interpolation operator Π_*h*_ is an integral average interpolation of *v* on the element *e*. So, in order to bound each term in the equation above

$$\begin{array}{c} \sum_{K}{\left(f-div\left(\beta u_{h}\right)-bu_{h},v-v_{h}\right)}_{K}\leq \sum_{K}Ch_{K}\|f-div\left(\beta u_{h}\right)-bu_{h}{\|}_{K}{\left\|v\right\|}_{H^{1}\left(K\right)}. \end{array}$$ (16)

$$\begin{array}{c} \int_{\partial K_{-}\backslash \Gamma_{-}}\left(\tau \left[u_{h}\right]\left[v_{h}-v\right]+\left(\beta \cdot n\right)\left[u_{h}\right]\left\{v-v_{h}\right\}\right)ds\leq \sum_{e\in \partial K_{-}\cap \Gamma_{-}}Ch_{e}^{\frac{1}{2}}\tau \|\left[u_{h}\right]{\|}_{L^{2}\left(e\right)}{\left\|v\right\|}_{H^{1}\left(K\right)}. \end{array}$$ (17)

$$\begin{array}{c} \begin{array}{cc} & \int_{\partial K_{+}\cap \Gamma_{+}}\left(\beta \cdot n\right)u_{h}\left(v_{h}-v\right)ds+\int_{\partial K_{-}\cap \Gamma_{-}}\left(\beta \cdot n\right)\left(g-u_{h}\right)\left(v_{h}-v\right)ds\\ & \leq \sum_{e\in \partial K_{+}\cap \Gamma_{+}}Ch_{e}^{\frac{1}{2}}\|u_{h}{\|}_{L^{2}\left(e\right)}{\left\|v\right\|}_{H^{1}\left(K\right)}+\sum_{e\in \partial K_{-}\cap \Gamma_{-}}Ch_{e}^{\frac{1}{2}}\|g-u_{h}{\|}_{L^{2}\left(e\right)}{\left\|v\right\|}_{H^{1}\left(K\right)}. \end{array} \end{array}$$ (18)

Using the definition of *L*^2^ norm and combing the above inequalities (16), (17) and (18), it is found that,

$$\begin{array}{ccc} \|\left(u-u_{h}\right){\|}_{L^{2}\left(\Omega \right)} & = & \underset{\nabla v\in L_{0}^{2}\left(\Omega \right)}{\text{sup}}\frac{\left(u-u_{h},\beta \nabla v\right)}{{\left\|\beta \nabla v\right\|}_{L^{2}\left(\Omega \right)}}\\ & \leq & \sum_{K}ch_{K}\|f-div\left(\beta u_{h}\right)-bu_{h}{\|}_{K}+\sum_{e\in \partial K_{-}\backslash \Gamma_{-}}Ch_{e}^{\frac{1}{2}}\tau \|\left[u_{h}\right]{\|}_{L^{2}\left(e\right)}\\ & & +\sum_{e\in \partial K_{+}\cap \Gamma_{+}}Ch_{e}^{\frac{1}{2}}\|u_{h}{\|}_{L^{2}\left(e\right)}{\left\|v\right\|}_{H^{1}\left(K\right)}\\ & & +\sum_{e\in \partial K_{-}\cap \Gamma_{-}}Ch_{e}^{\frac{1}{2}}\|g-u_{h}{\|}_{L^{2}\left(e\right)}{\left\|v\right\|}_{H^{1}\left(K\right)}. \end{array}$$

Here, assume that the vector function *β* has the lower bound, i.e., min||*β*|| ≥ *c*_0_ > 0. Therefore,

$$\begin{array}{c} \begin{array}{cc} C_{0}\|u-u_{h}{\|}_{L^{2}\left(\Omega \right)} & \leq \sum_{K}Ch_{K}\|f-div\left(\beta u_{h}\right)-bu_{h}{\|}_{K}+\sum_{e\in \partial K_{-}\backslash \Gamma_{-}}Ch_{e}^{\frac{1}{2}}\tau \|\left[u_{h}\right]{\|}_{L^{2}\left(e\right)}\\ & +\sum_{e\in \partial K_{+}\cap \Gamma_{+}}Ch_{e}^{\frac{1}{2}}\|u_{h}{\|}_{L^{2}\left(e\right)}{\left\|v\right\|}_{H^{1}\left(K\right)}\\ & +\sum_{e\in \partial K_{-}\cap \Gamma_{-}}Ch_{e}^{\frac{1}{2}}\|g-u_{h}{\|}_{L^{2}\left(e\right)}{\left\|v\right\|}_{H^{1}\left(K\right)}. \end{array} \end{array}$$ (19)

From the above equations, it can be calculated that,

$$\begin{array}{c} \begin{array}{cc} \|u-u_{h}{\|}_{L^{2}\left(\Omega \right)} & \leq \sum_{K}Ch_{K}\|f-div\left(\beta u_{h}\right)-bu_{h}{\|}_{L^{2}\left(K\right)}+\sum_{e\in \partial K_{-}\backslash \Gamma_{-}}Ch_{e}^{\frac{1}{2}}\tau \|\left[u_{h}\right]{\|}_{L^{2}\left(e\right)}\\ & +\sum_{e\in \partial K_{+}\cap \Gamma_{+}}Ch_{e}^{\frac{1}{2}}\|u_{h}{\|}_{L^{2}\left(e\right)}+\sum_{e\in \partial K_{-}\cap \Gamma_{-}}Ch_{e}^{\frac{1}{2}}\|g-u_{h}{\|}_{L^{2}\left(e\right)}. \end{array} \end{array}$$

So, by inserting *η*, the proof of the theorem is completed.

**Theorem 4.2** *Let u and u*_*h*_ ∈ *V*_*h*_ *be the finite element solutions of* (1) *and* (5), *respectively. For any* 0 < *e* ≤ *K* *and* *h* > 0, *there is a constant C which is dependent on* $T_{h}$ *but independent of u*, *e* *and* *h*. *Then for the lower bound the following error representation formula holds*:

$$\begin{array}{c} \|u-u_{h}{\|}_{L^{2}\left(\Omega \right)}\geq C\eta . \end{array}$$

*Proof*: Using integration by parts,

$$\begin{array}{ccc} \int_{K}\left(u-u_{h}\right)\beta \nabla vdx & = & \int_{\partial K}\beta n\left(u-u_{h}\right)vds-\int_{K}div\left(\beta \left(u-u_{h}\right)\right)vdx\\ & = & \int_{\partial K}\beta n\left(u-u_{h}\right)vds-\int_{K}\left(f-div\left(\beta u_{h}\right)\right)vdx. \end{array}$$

For our problem (1), it can be rewritten as

$$\begin{array}{c} \int_{K}\left(u-u_{h}\right)\beta \nabla vdx=\int_{\partial K}\beta n\left(u-u_{h}\right)vds-\int_{K}\left(f-div\left(\beta u_{h}\right)-bu_{h}\right)vdx. \end{array}$$

Taking *v* = *b*_*K*_(*f* − *div*(*βu*_*h*_) − *bu*_*h*_) in the above equation, where *b*_*K*_ is the hat function with the support set *K*.

$$\begin{array}{ccc} C\|f-div\left(\beta u_{h}\right)-bu_{h}{\|}_{K}^{2} & \leq & \|b_{K}^{\frac{1}{2}}\left(f-div\left(\beta u_{h}\right)-bu_{h}\right){\|}_{K}^{2}\\ & = & \int_{K}\left(u-u_{h}\right)\beta \nabla \left(b_{K}\left(f-div\left(\beta u_{h}\right)-bu_{h}\right)\right)dx\\ & \leq & \|u-u_{h}{\|}_{K}\|\nabla \left(b_{K}\left(f-div\left(\beta u_{h}\right)-bu_{h}\right)\right){\|}_{K}\left\|\beta \right\|\\ & \leq & Ch_{K}^{-1}\|u-u_{h}{\|}_{K}\|f-div\left(\beta u_{h}\right)-bu_{h}{\|}_{K}. \end{array}$$

So we obtain

$$\begin{array}{cc} C\|f-div\left(\beta u_{h}\right)-bu_{h}{\|}_{K}\leq & Ch_{K}^{-1}\|u-u_{h}{\|}_{K}. \end{array}$$ (20)

Taking *v* = *b*_*e*_*P*_*r*_((*β* ⋅ **n**)[*u*_*h*_]), where *P*_*r*_ is the extend operator. *v* = *b*_*e*_*Pr*((*β* ⋅ **n**)[*u*_*h*_]) satisfies

$$\begin{array}{ccc} \|b_{e}P_{r}\left(\left[u_{h}\right]\right){\|}_{K} & \leq & C\sqrt{\left|e\right|}\|\left[u_{h}\right]{\|}_{e}\\ \|\nabla \left(b_{e}P_{r}\left(\left[u_{h}\right]\right)\right){\|}_{K} & \leq & Ch_{K}^{-1}\|b_{e}P_{r}\left(\left[u_{h}\right]\right){]\|}_{K}\leq C_{2}h_{K}^{-1}\sqrt{\left|e\right|}\|\left[u_{h}\right]{\|}_{e}. \end{array}$$

Using above inqualties in order to obtain

$$\begin{array}{ccc} C\sqrt{\left|e\right|}\|\left(\beta \cdot n\right)\left[u_{h}\right]{\|}_{e}^{2} & \leq & \int_{e}\left(\beta \cdot n\right)\left[u_{h}\right]b_{e}Pr\left(\left(\beta \cdot n\right)\left[u_{h}\right]\right)ds\\ & = & \int_{K}\left(f-div\left(\beta u_{h}\right)-bu_{h}\right)b_{e}P_{r}\left(\left[u_{h}\right]\right)dx\\ & + & \int_{K}\left(u-u_{h}\right)\beta \nabla \left(b_{e}P_{r}\left(\left[u_{h}\right]\right)\right)dx\\ & \leq & Ch_{K}^{-1}\|u-u_{h}{\|}_{K}\sqrt{\left|e\right|}\|\left(\beta \cdot n\right)\left[u_{h}\right]{\|}_{e}. \end{array}$$

Therefore,

$$\begin{array}{c} \|\left(\beta \cdot n\right)\left[u_{h}\right]{\|}_{e}\leq Ch_{e}^{-\frac{1}{2}}\|u-u_{h}{\|}_{K}. \end{array}$$ (21)

Now, combining the Eqs (19), (20) and (21), it can be found that,

$$\begin{array}{c} \begin{array}{cc} & \sum_{K}Ch_{K}\|f-div\left(\beta u_{h}\right)-bu_{h}{\|}_{L^{2}\left(K\right)}+\sum_{e\in \partial K_{-}\backslash \Gamma_{-}}Ch_{e}^{\frac{1}{2}}\tau \|\left[u_{h}\right]{\|}_{L^{2}\left(e\right)}\\ & +\sum_{e\in \partial K_{+}\cap \Gamma_{+}}Ch_{e}^{\frac{1}{2}}\|u_{h}{\|}_{L^{2}\left(e\right)}+\sum_{e\in \partial K_{-}\cap \Gamma_{-}}Ch_{e}^{\frac{1}{2}}\|g-u_{h}{\|}_{L^{2}\left(e\right)}\leq \|u-u_{h}{\|}_{L^{2}\left(\Omega \right)}. \end{array} \end{array}$$

Again, insert the values of *η*, the proof of the theorem is completed.

## 5 Numerical experiments

In this section, numerical experiments are presented to validate the theoretical properties which are obtained in this article for the first order hyperbolic equation. The mesh generation and all computations are done by FreeFem++ [33]. The algorithm of (5) is executed on the uniform triangular mesh sequence to authenticate the theoretical results. The discrete space *V*_*h*_ is constructed by using piecewise polynomials of uniformed degree. The values of *β*, *b*, *θ*, and *γ* are specified for the setting of the proper function *f* and exact solution *u*(*x*, *y*).

### 5.1 Experiments for a priori error estimate

For the a priori error analysis, first four numerical experiments are presented here. The error profiles and corresponding convergence rates are given in Tables 1–4 against the mesh-size. In all numerical experiments, the convergence behavior of errors ‖*u* − *u*_*h*_‖ with respect to the weighed parameter *θ* and penalty parameter *γ* are presented, respectively on uniform triangular meshes. The boundary condition and the proper functions *f* and *g* are chosen such that *u*(*x*, *y*) is the exact solution. Consider the domain Ω = (0, 1)^2^ for all numerical tests.

**Table 1:** Error profile and convergence rate for the Experiment 1.

*Mesh*	‖*u* − *u*_*h*_‖	*Order*	*Mesh*	‖*u* − *u*_*h*_‖	*Order*
4	0.000204707		32	3.02978e-006	2.05828
8	5.01865e-005	2.02819	64	1.69708e-006	1.95802
16	1.26187e-005	1.99174	128	9.72978e-007	1.98128

**Table 2:** Error profile and convergence rate for the Experiment 2.

*Mesh*	‖*u* − *u*_*h*_‖	*Order*	*Mesh*	‖*u* − *u*_*h*_‖	*Order*
4	0.000906352		32	1.41629e-005	1.99424
8	0.000225648	2.0060	64	9.84192e-006	1.92433
16	5.6426e-005	1.99964	128	7.91416e-006	1.91942

**Table 3:** Error profile and convergence rate for the Experiment 3.

*Mesh*	‖*u* − *u*_*h*_‖	*Order*	*Mesh*	‖*u* − *u*_*h*_‖	*Order*
4	0.0116658		32	0.000135048	1.9520
8	0.00217739	2.42162	64	0.000095043	1.9652
16	0.000522516	2.05905	128	0.000069355	1.9805

**Table 4:** Error profile and convergence rate for the Experiment 4.

*Mesh*	‖*u* − *u*_*h*_‖	*Order*	*Mesh*	‖*u* − *u*_*h*_‖	*Order*
4	0.000739568		32	2.90955e-006	1.81876
8	0.000547261	0.43445	64	1.01505e-006	1.88187
16	0.000102642	2.41460	128	9.60955e-007	1.98108

### 5.2 Experiment 1

The first experiment is obtained by choosing data functions *g*, *β* = (2 − *y*^2^, 2 − *x*)^*T*^ and *f* = 6 + *x* + 6*y* − *y*^2^ so that the exact solution is *u*(*x*, *y*) = *x* + 2*y* on the domain. The stability parameters are *θ* = 500, *γ* = 3, *b* = 3.

### 5.3 Experiment 2

For the second experiment we obtained data functions as, *g*, *β* = (2 − *y*, 2 − *x*)^*T*^ and $f=\left(2x-3\right)ye^{-x-y^{2}}$ so that the exact solution is $u\left(x,y\right)=e^{-x-y^{2}}$ on the domain. The stability parameters are *θ* = 100, *γ* = 4, *b* = 2.

### 5.4 Experiment 3

The third experiment is considered by choosing data functions *g*, *β* = (−*y*, *x*)^*T*^ and *f* = 2*sin*(*πx*)*sin*(*πy*) + *πxsin*(*πx*)*cos*(*πy*) − *πysin*(*πy*)*cos*(*πx*) so that the exact solution is *u*(*x*, *y*) = *sin*(*πx*)*sin*(*πy*) on the domain. The stability parameters are *θ* = 300, *γ* = 5, *b* = 2.

### 5.5 Experiment 4

The fourth experiment’s data functions are chosen as *g*, *β* = (1, 2)^*T*^ and *f* = 2*x* + *y* − 2*x*^2^ − *y*^2^ − 5*xy* + 3*x*^2^*y* + *xy*^2^ + *x*^2^*y*^2^ so that the exact solution is *u*(*x*, *y*) = *x*(*x* − 1)*y*(*y* − 1) on the domain. The stability parameters are *θ* = 1000, *γ* = 2, *b* = 1.

**Fig 1:**
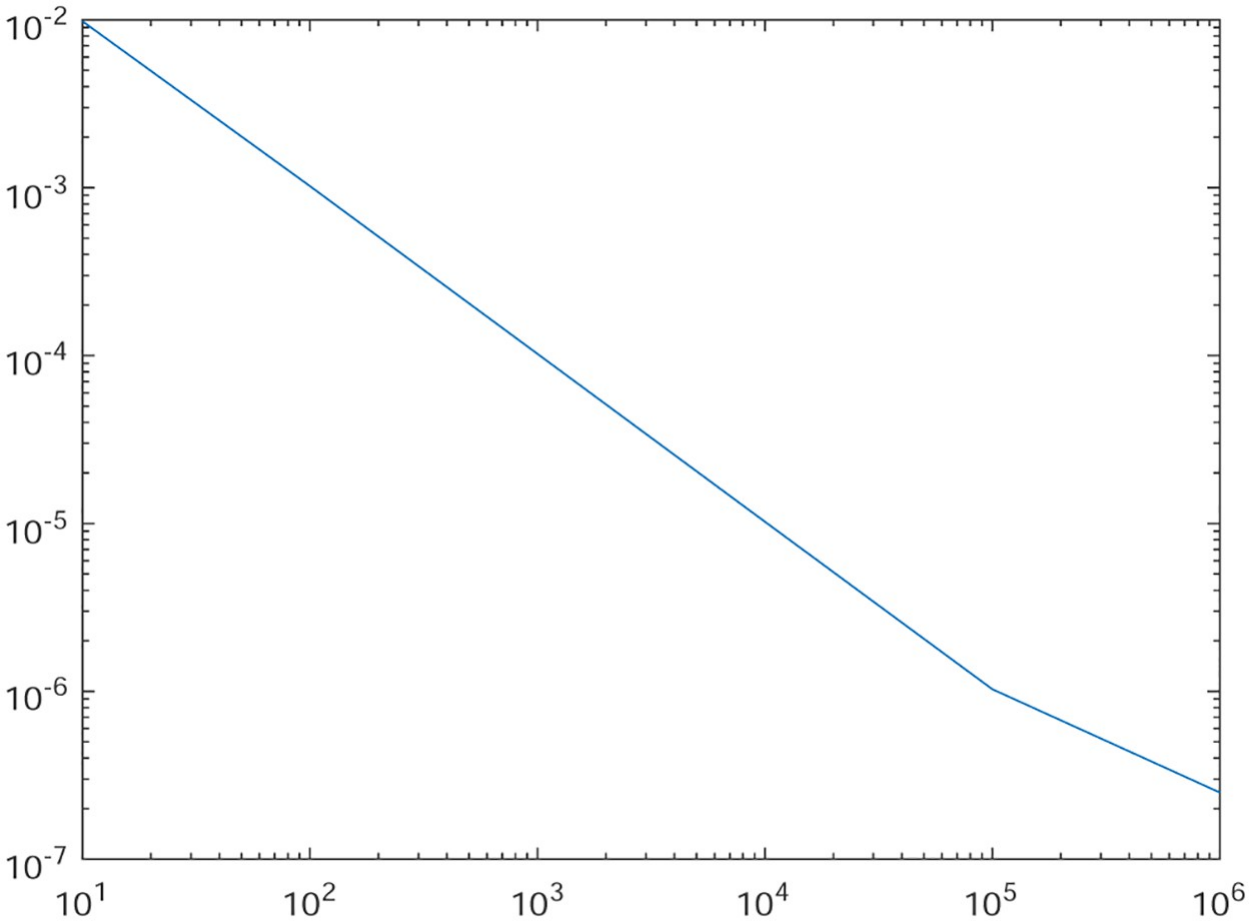
The behavior of the error with respect to the parameter *θ* for the Experiment 1.

**Fig 2:**
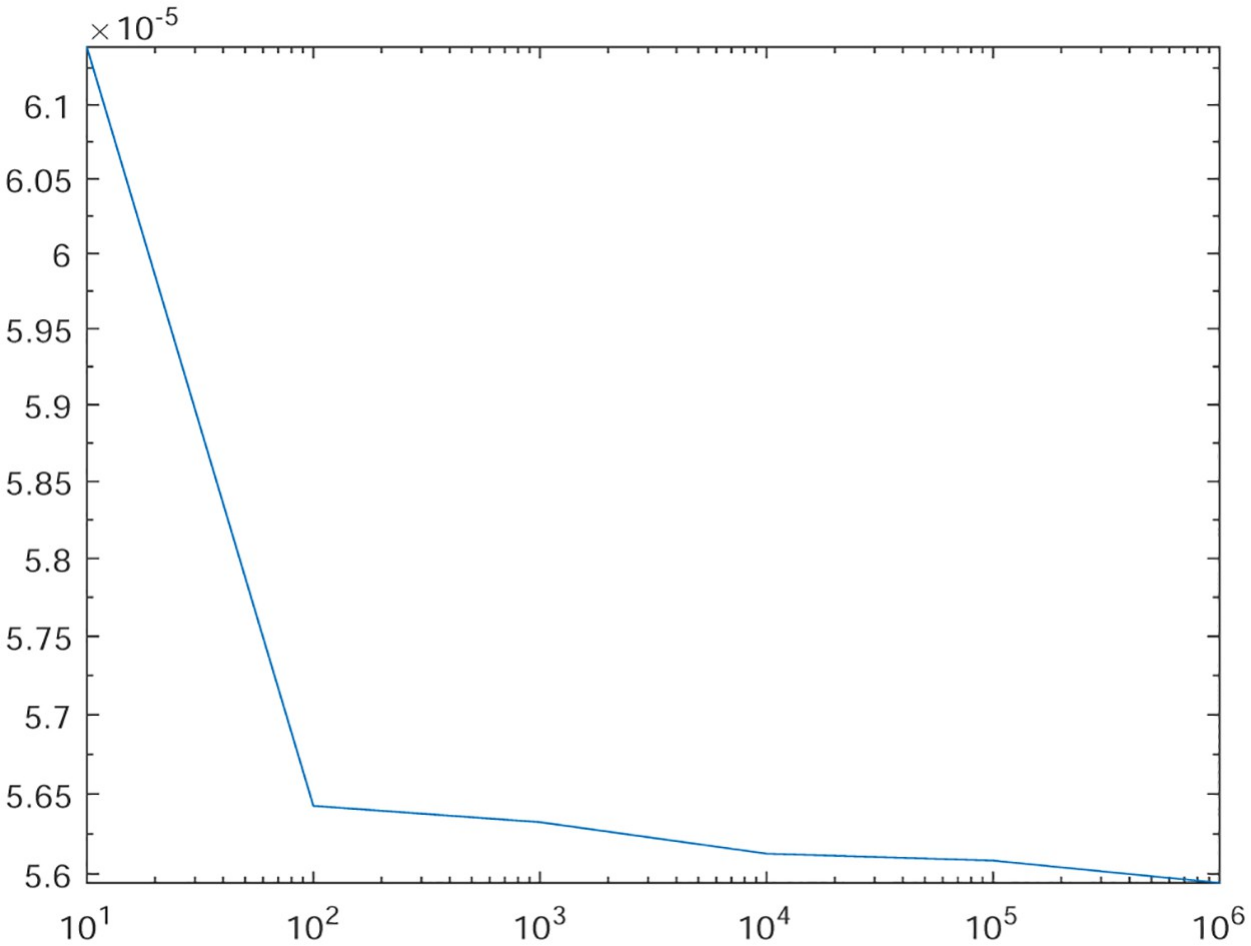
The behavior of the error with respect to the parameter *θ* for the Experiment 2.

**Fig 3:**
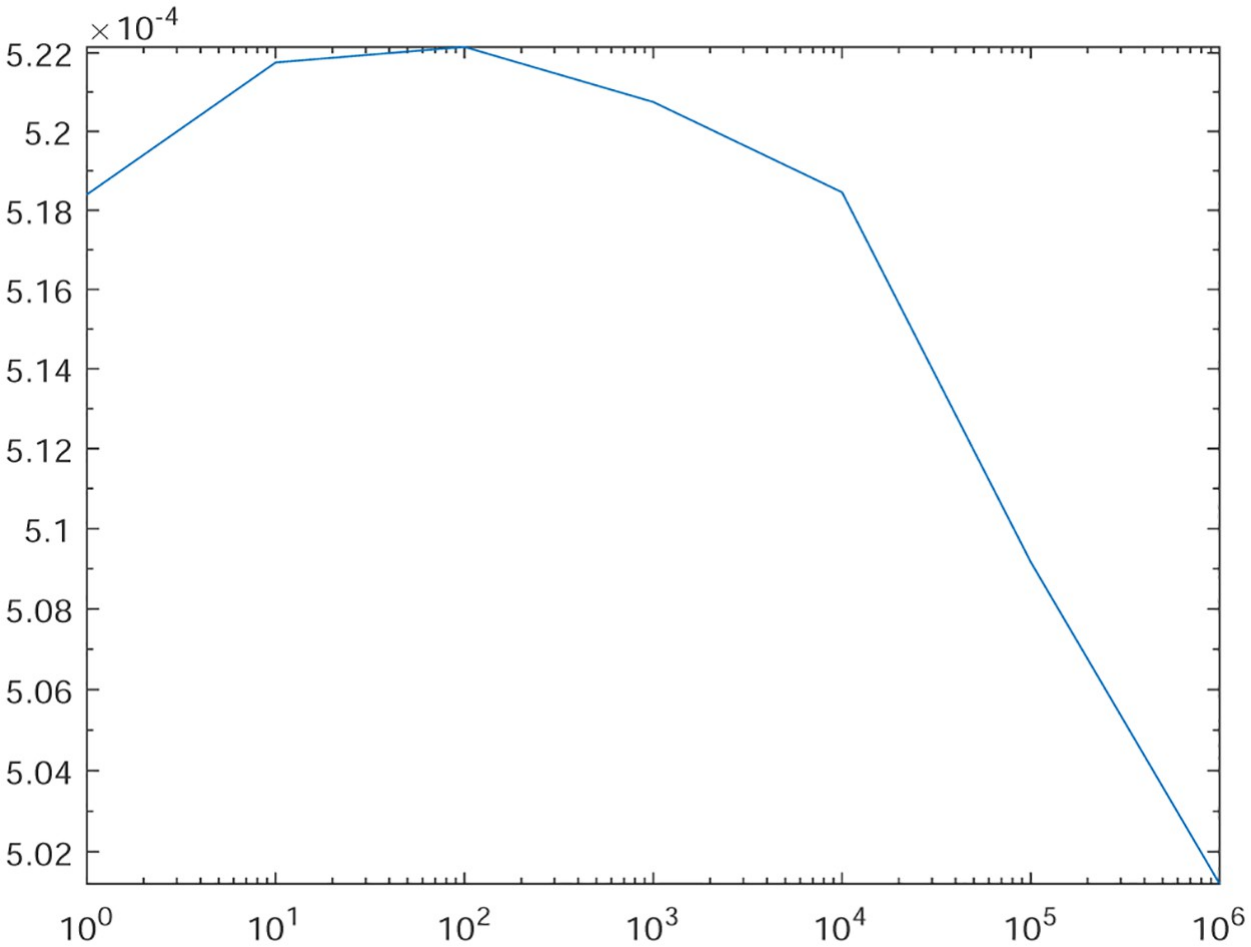
The behavior of the error with respect to the parameter *θ* for the Experiment 3.

**Fig 4:**
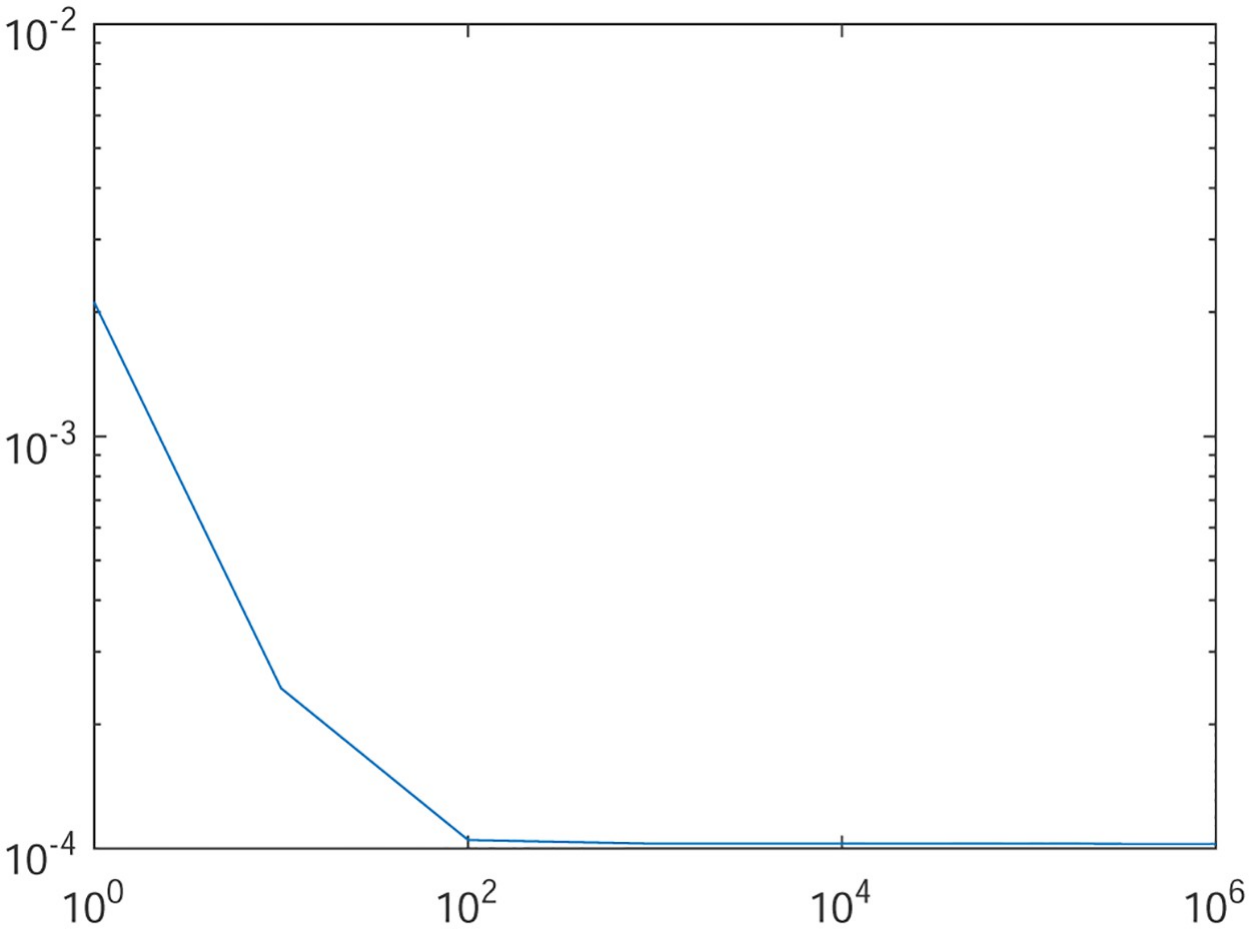
The behavior of the error with respect to the parameter *θ* for the Experiment 4.

**Fig 5:**
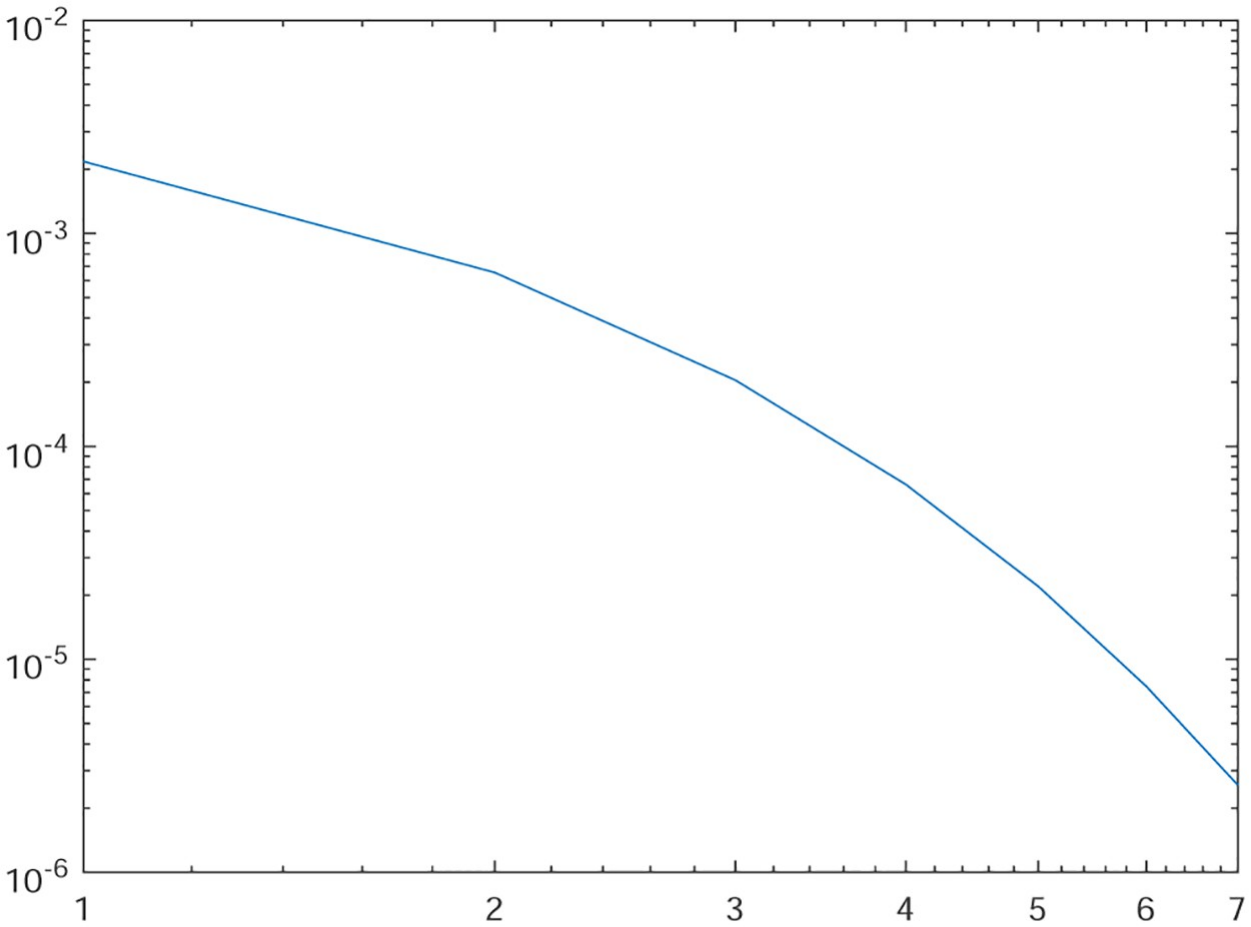
The behavior of the error with respect to the parameter *γ* for the Experiment 1.

**Fig 6:**
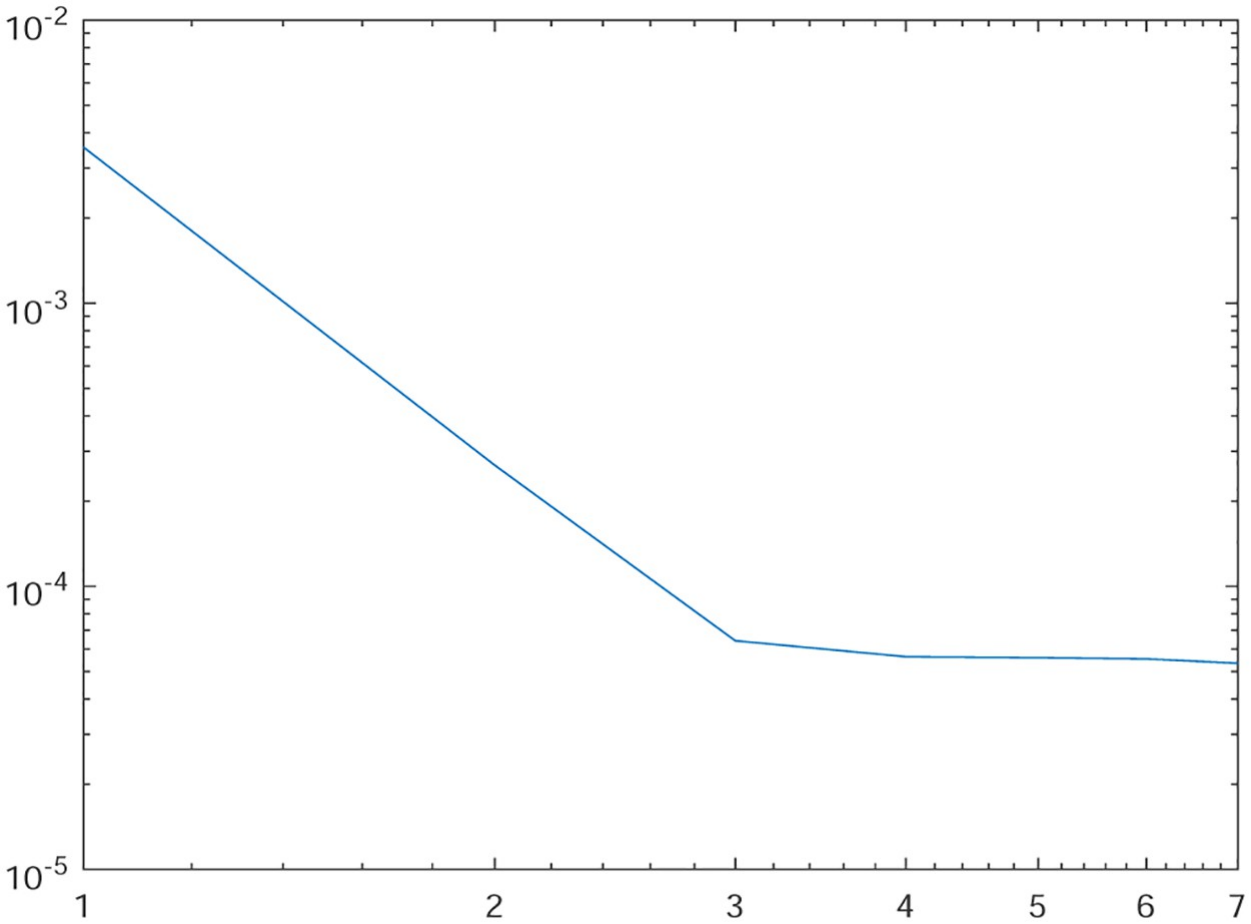
The behavior of the error with respect to the parameter *γ* for the Experiment 2.

**Fig 7:**
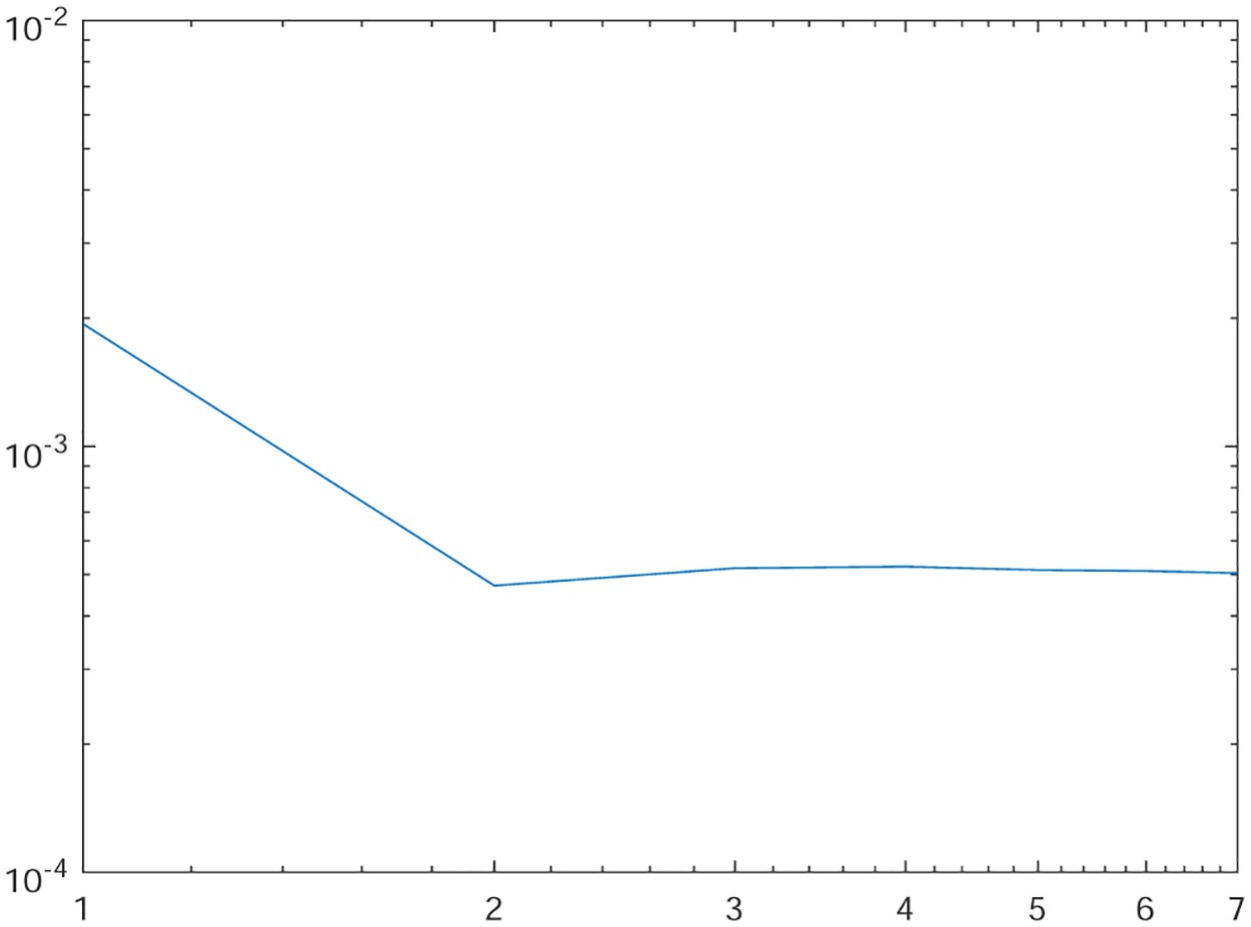
The behavior of the error with respect to the parameter *γ* for the Experiment 3.

**Fig 8:**
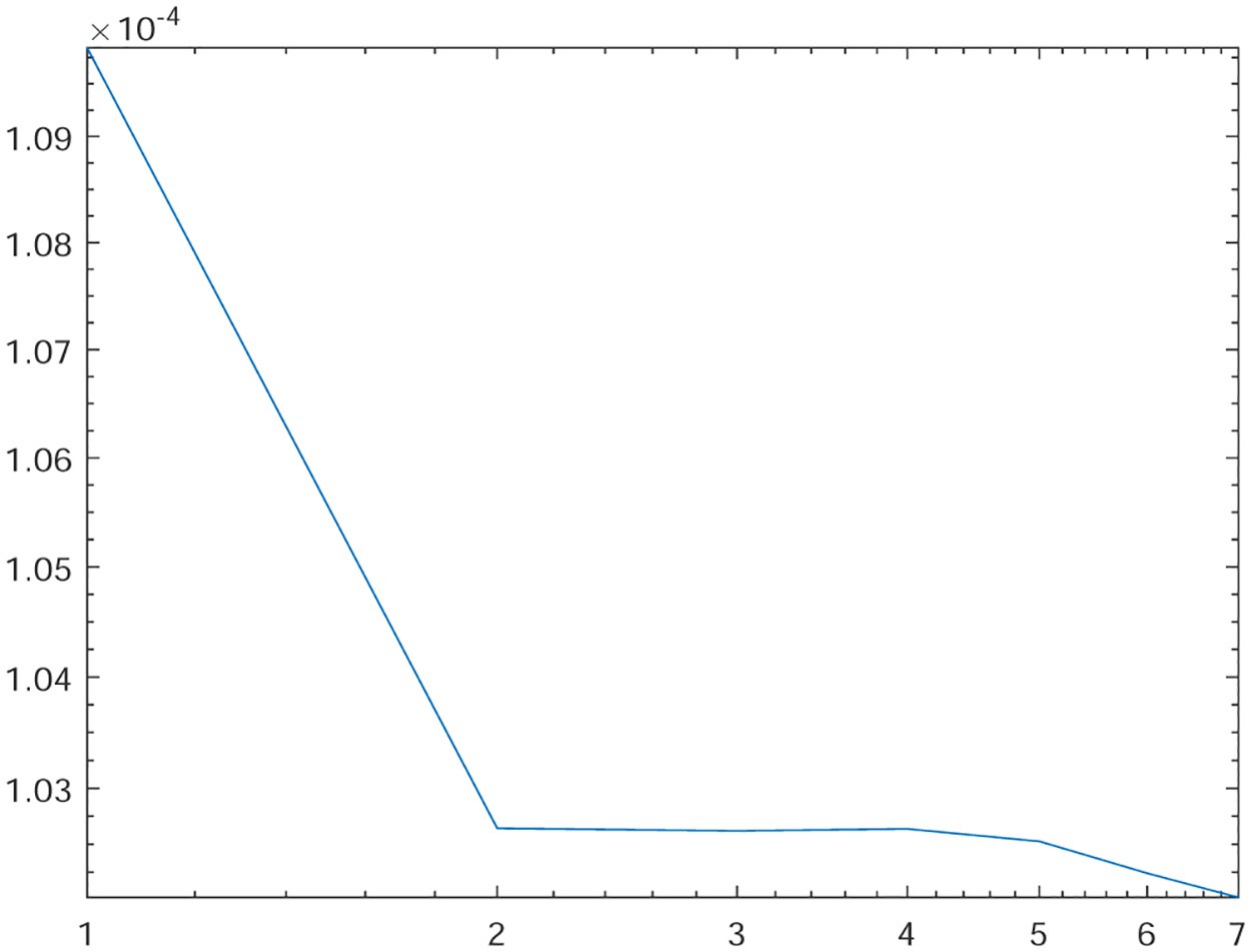
The behavior of the error with respect to the parameter *γ* for the Experiment 4.

Tables 1–4 present the *L*^2^-norm of the error ‖*u* − *u*_*h*_‖ and order of convergence against the meshes at 4, 8, 16, 32, 64, 128,respectively. From all the Tables 1–4, it is observed that the quantity ‖*u* − *u*_*h*_‖ have the convergence rates as predicted by the theorem and the computed order of convergence is more than 2. For all the four experiments, the influence of varying *θ* are demonstrated at a certain value of *γ* = 5 and mesh at 32 on the error in Figs 1–4, respectively. The values of *θ* are taken from 10 to 10^6^ for the experiments. From these figures, it is found that, varying *θ* seems to have more influence on the error than varying *h*. On the other hand, the influence of varying *γ* are established in Figs 5–8 on the error, respectively. At this time, the values of *γ* are considered from 1 to 7 at a fixed value of *θ* = 100 and mesh at 16. From these figures, it is also found that, varying *γ* has a significant influence on the error.

### 5.6 Experiments for a posteriori error estimate

For the a posteriori error estimate, two numerical experiments are demonstrated here. The residual-based a posteriori error estimation method is applied. Numerical examples are presented in a square and an L-shaped domain. For the mesh adaption and error estimates, consider the residual error indicator *η* on the element *K* for the problem.

### 5.7 Experiment 5

**Fig 9:**
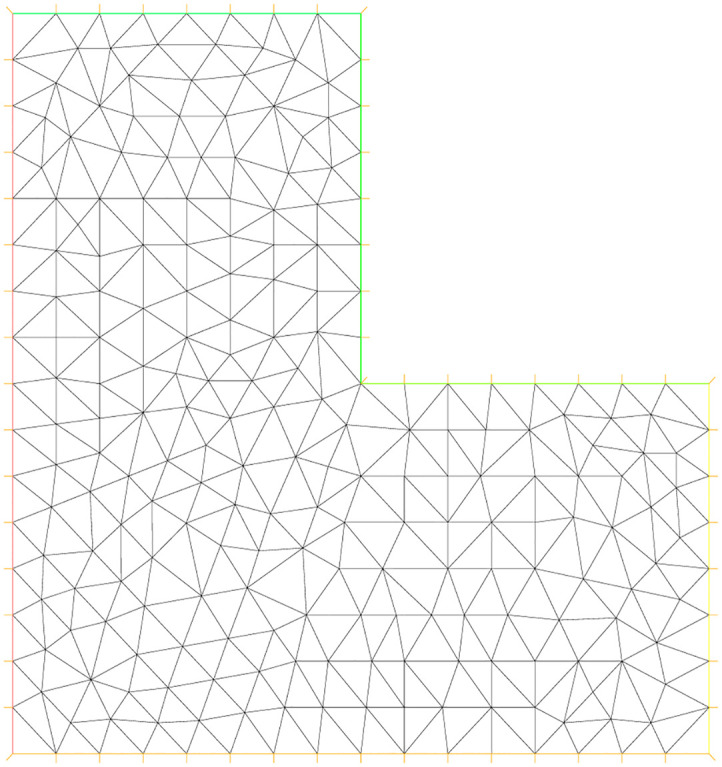
Initial adaptive mesh refinement for the L-shaped domain.

**Fig 10:**
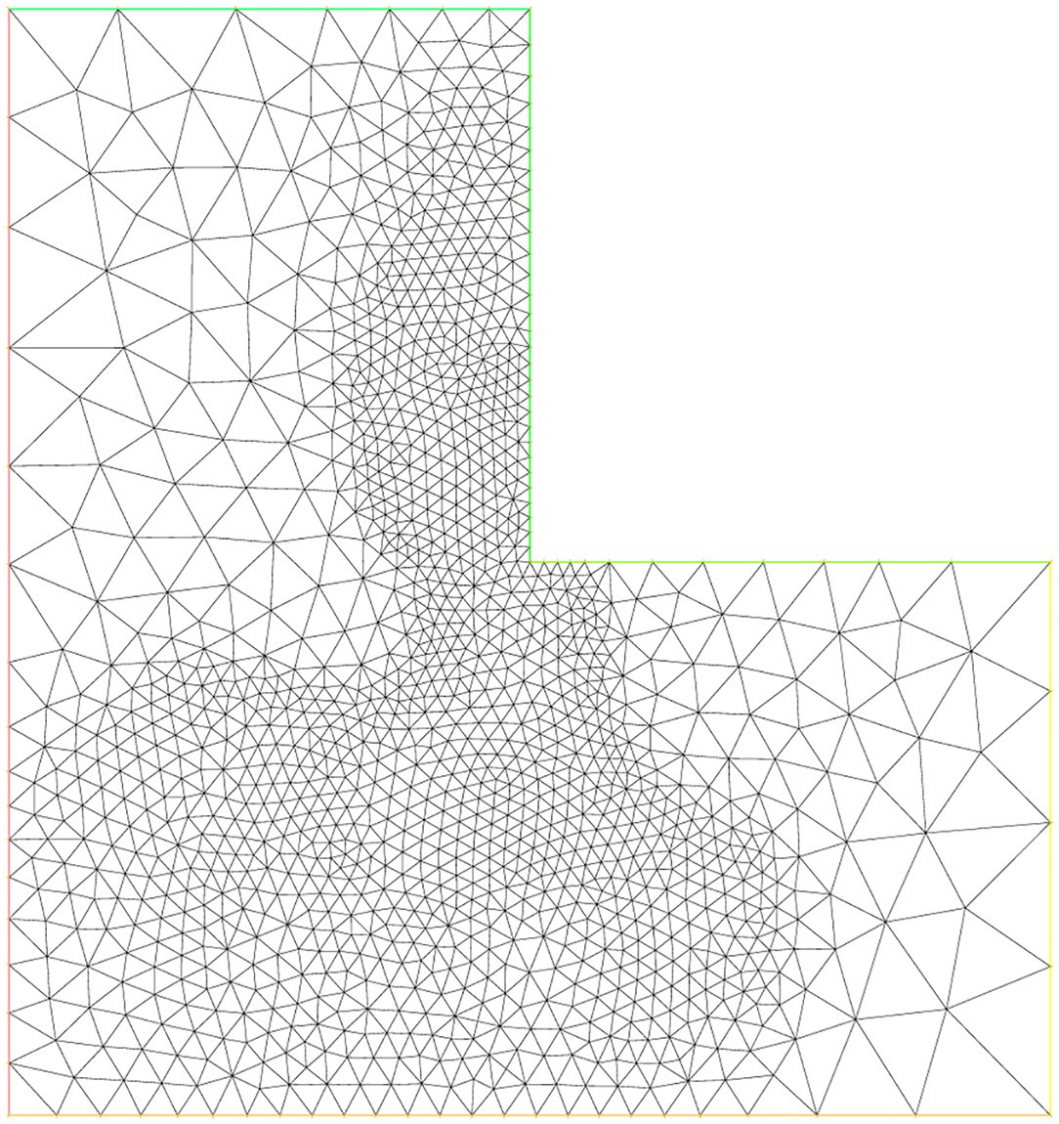
Adaptive mesh refinement with 4899 nodes for the L-shaped domain.

**Fig 11:**
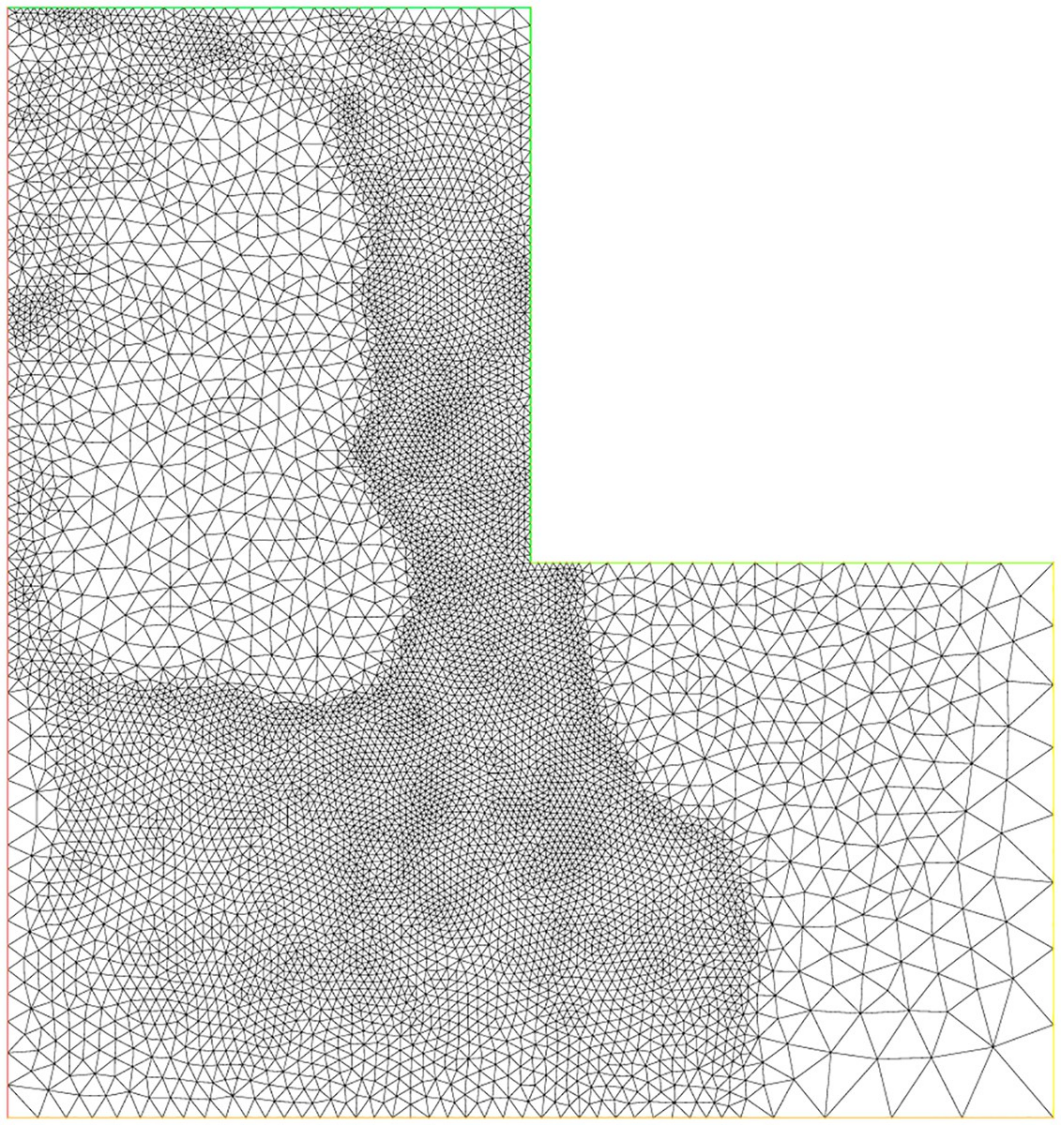
Adaptive mesh refinement with 13804 nodes for the L-shaped domain.

For this experiment, the function *f*, the exact solution *u*(*x*, *y*) and all parameters are same as are given in experiment 3. In this example, the L-shaped domain Ω is considered, which has vertices (0, 0), (1, 0), (0, 1), (−1, 1), (−1, −1) and (1, −1). Figs 9–11 correspond to the sequence of adaptively refined meshes and Table 5 displays the numerical results for the meshes generated by the mesh-refining Algorithm. The number of degree of freedom *N*, the corresponding relative error ‖*u* − *u*_*h*_‖ in the *L*^2^(Ω)-norm and the residual error indicator *η* are shown in Table 5 for experiment 5.

**Table 5:** Errors ‖*u* − *u*_*h*_‖_*L*^2^_ and error indicator *η* of the L-shape mesh for experiment 5.

*N*	‖*u* − *u*_*h*_‖_*L*^2^_	*η*	*N*	‖*u* − *u*_*h*_‖_*L*^2^_	*η*
183	5.133117e-001	4.02913e-000	4899	1.377801e-003	7.87314e-003
316	1.137110e-001	6.49777e-001	8674	9.285120e-004	5.03258e-003
621	9.877859e-002	5.39184e-001	11407	4.321520e-005	2.32338e-004
1197	2.842097e-002	1.57456e-001	13804	2.954530e-005	1.57994e-004
2523	4.211333e-003	2.28876e-002	19712	9.934530e-006	5.32682e-005

### 5.8 Experiment 6

A compressible hyperbolic problem is considered here with *β* = (2*y*^2^ − 4*x* + 1, 1 + *y*)^*T*^, *b* = 0, the stability parameters *θ* = 1000 and *γ* = 1, respectively. For this experiment, the unit square Ω = (0, 1)^2^ domain is taken for the adaptive mesh refinement. The function *f* and the boundary condition *g* are chosen as like:

$$u\left(x,y\right)=\{\begin{array}{cc} 0 & \text{for}\;x=0,\;0.5<y\leq 1,\\ 1 & \text{for}\;x=0,\;0\leq y\leq 0.5,\\ 1 & \text{for}\;0\leq x\leq 0.75,\;y=0,\\ 0 & \text{for}\;0.75\leq x\leq 1,\;y=0,\\ sin^{2}\left(\pi y\right) & \text{for}\;x=1,\;0\leq y\leq 1. \end{array}$$

**Fig 12:**
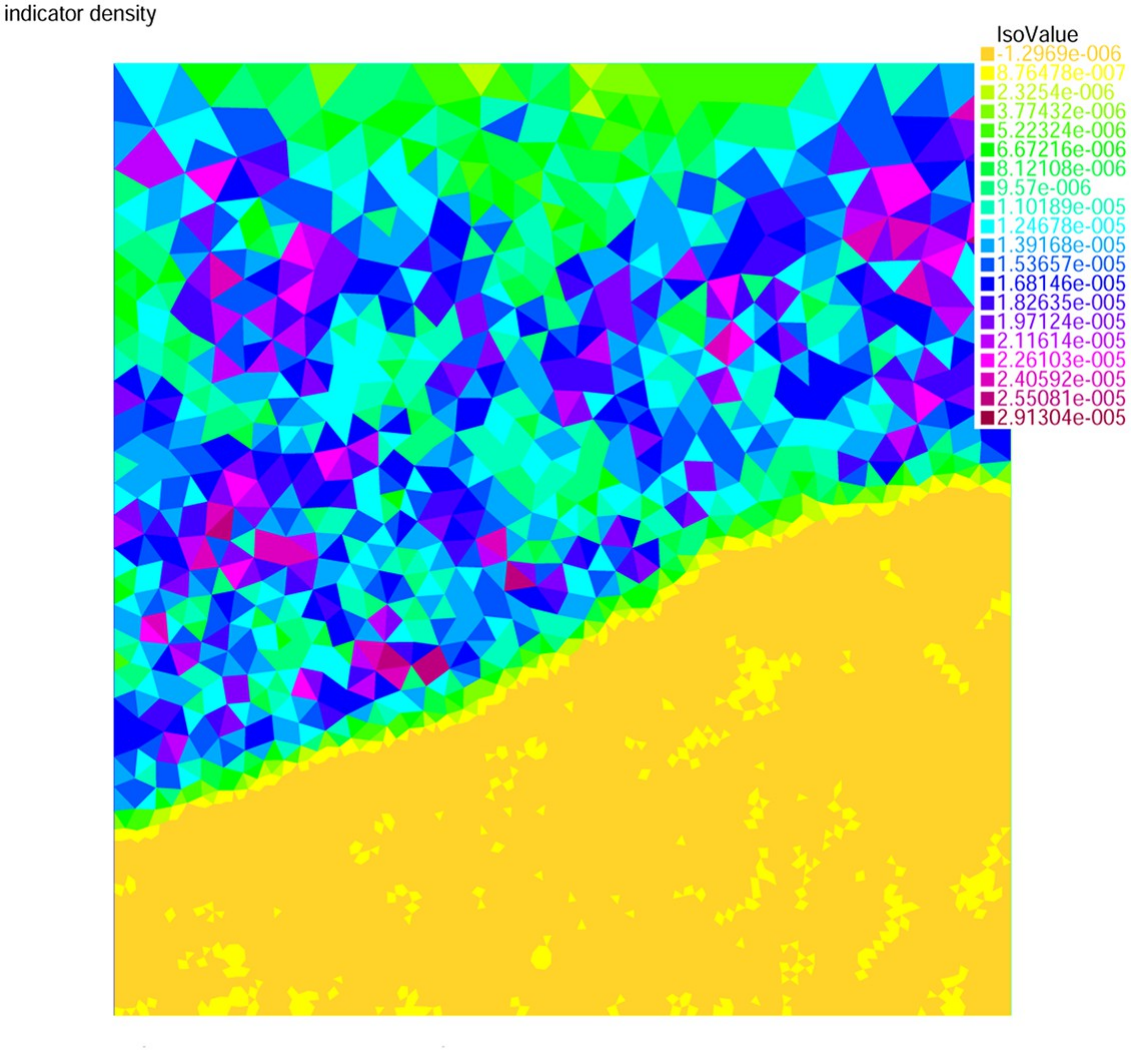
Density of error indicator for experiment 6.

**Fig 13:**
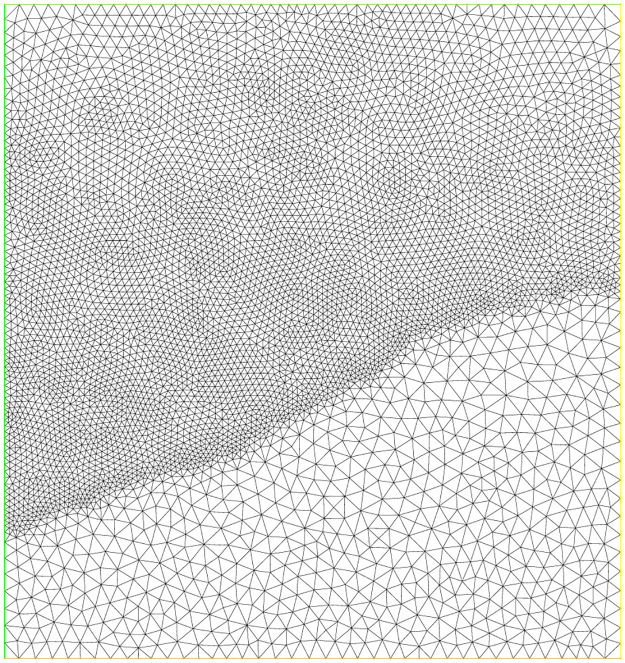
Adaptive mesh refinement with 19670 nodes for experiment 6.

In this example, Fig 12 presents the residual error indicator and Fig 13 represents the adaptive mesh refinement with 19670 nodes. The number of degree of freedom *N*, the corresponding relative error ‖*u* − *u*_*h*_‖ in the *L*^2^(Ω)-norm and the residual error indicator *η* are presented in Table 6 for experiment 6. From this two numerical experiments, It is deduced that, the adaptive mesh refining method is more powerful tools for the hyperbolic problems, especially on the boundary elements. The convergence rates are improved to the optimal order and the error estimates are efficient and stout.

**Table 6:** Errors ‖*u* − *u*_*h*_‖_*L*^2^_ and error indicator *η* of the unit square mesh for experiment 6.

*N*	‖*u* − *u*_*h*_‖_*L*^2^_	*η*	*N*	‖*u* − *u*_*h*_‖_*L*^2^_	*η*
181	0.000793318	0.001913740	10694	2.133042e-006	0.000054372
682	0.000127386	0.000838299	13130	1.587852e-006	0.000044299
1341	4.597850e-005	0.000395739	15401	1.264791e-006	0.000037723
3025	1.372830e-005	0.000183320	18135	1.007413e-006	0.000028571
6579	4.126251e-006	0.000078993	19670	7.995001e-007	0.000024825

## 6 Conclusion

In this article, a discontinuous Galerkin method have developed with a weighted parameter *θ* and a penalty parameter *γ* for the error estimation of first order hyperbolic equation. Numerical experiments have been presented which are evidently emphasized on the efficiency of both parameters on the optimal order of convergence of the solutions for both a priori and a posteriori error analysis. The method used in this paper can also be extended to the higher-order and to time dependent nonlinear hyperbolic problems to obtain the error estimates of those problems.
